# Telemedicine and the Attractiveness of a Family Medicine Career Among Hungarian Medical Students: Implications for the Digital Health Primary Care Workforce

**DOI:** 10.3390/healthcare14193256

**Published:** 2026-10-01

**Authors:** Viktor Rekenyi, Csongor István Szepesi, Nóra Horváth, László Róbert Kolozsvári

**Affiliations:** 1 Department of Family and Occupational Medicine, Faculty of Medicine, University of Debrecen, 4032 Debrecen, Hungary; 2 Doctoral School of Health Sciences, University of Debrecen, 4032 Debrecen, Hungary

**Keywords:** telemedicine, primary care informatics, digital health, medical students, general practice, career choice, health workforce, medical education

## Abstract

**Highlights:**

**What are the main findings?**

- Awareness of telemedicine among fifth-year medical students was near-universal, yet self-perceived knowledge was modest and showed no evidence of an association with how useful they rated telemedicine.
- Only about one-third viewed telemedicine as making general practice work more attractive; this perception was driven by pre-existing career interest and by the perceived usefulness of telemedicine, not by demographics.

**What are the implications of the main findings?**

- Telemedicine appears to reinforce, rather than independently create, interest in a family medicine career, so it should complement broader workforce measures.
- Integrating telemedicine competencies into the medical curriculum is needed so that positive attitudes are based on demonstrable skills rather than diffuse familiarity.

**Abstract:**

Background/Objectives: Telemedicine expanded rapidly during the COVID-19 pandemic and is now embedded in primary care, a field facing an acute workforce crisis. Whether it could make a general practitioner (GP) career more attractive to future physicians has largely been unexplored. We assessed fifth-year medical students’ perceptions of telemedicine and factors associated with viewing it as increasing the attractiveness of GP work. Methods: In this cross-sectional study, 140 of 154 fifth-year students at the University of Debrecen, Hungary (response rate 91%), completed a self-developed, paper-based questionnaire in early 2026. Ordinal data were analysed with non-parametric tests, a composite perceived usefulness score, and binary logistic regression, with Holm–Bonferroni correction for pre-specified comparisons. Results: Awareness was near-universal (98.6%), but self-perceived knowledge was modest and showed no association with usefulness ratings (rho = 0.024, 95% CI −0.14 to 0.19). Synchronous and clinical data modalities were rated more useful than informal text channels, and 82.9% preferred hybrid working. Only 32.1% thought telemedicine would make GP work more attractive; this perception was independently associated with GP career interest (adjusted OR 2.59, 95% CI 1.47–4.57) but not with sex, age, or knowledge. Conclusions: Telemedicine is viewed as useful but appears to reinforce existing career interest rather than independently attract students to general practice.

## 1. Introduction

Telemedicine, defined by the World Health Organization as the delivery of health-care services at a distance through information and communication technologies, is intended to improve clinical management and to extend the reach of care [1]. It is not a single technology but a family of modalities conventionally grouped into real-time (synchronous) interaction, store-and-forward (asynchronous) exchange, and remote patient monitoring, delivered over channels that range from the telephone to dedicated video and application-based platforms [2]. For the present study, telemedicine is understood as clinical interaction between a clinician and a patient (or between clinicians) conducted at a distance for diagnosis, treatment, monitoring, or advice, which distinguishes it from the general, non-clinical use of the same digital tools, and it is one of the most patient-facing components of primary care informatics. Its diffusion, long gradual, was abruptly accelerated by the COVID-19 pandemic, when remote care became a principal means of maintaining continuity while reducing the risk of viral transmission [3]; this expansion did not recede afterwards, and telemedicine is now an established part of outpatient and primary care, even as questions of quality, digital literacy, and equitable access remain [4]. In Hungary, the same shift was accompanied by a rapid regulatory response that established the legal basis for telemedicine, notably Government Decree 157/2020 (IV. 29.), commonly termed the Telemedicine Decree, together with Act LVIII of 2020 (Section 37) and Decree 33/2020 (IX. 16.) of the Ministry of Human Capacities [5,6,7,8]. In routine Hungarian primary care, telemedicine is now used chiefly for electronic prescribing, telephone and video consultation, and the remote monitoring of chronic conditions, and population-level adoption rose markedly between 2021 and 2024 [9,10], although physicians remain more receptive to tools that support communication with patients than to those that support clinical work [9].

General practitioners (in Hungary, haziorvosok) are, in the European definition, specialist physicians who act as the point of first medical contact and provide comprehensive and continuing care to every individual irrespective of age, sex, or illness [11]; in Hungary, family medicine is a recognised specialty entered through a dedicated residency, and each practice serves a defined, registered patient list. Primary care of this kind is widely regarded as the foundation of resilient health systems, yet it faces a deepening workforce crisis. The global health workforce shortage, although projected to fall from about 15 million in 2020 to roughly 10 million by 2030, remains acute and inequitably distributed [12], and shortages and recruitment difficulties in primary care are reported across the European Union and neighbouring countries [13]. The strain is compounded by high rates of burnout among general practitioners (GPs), whose pooled global prevalence is approximately one-third [14]. Hungary exemplifies these trends: over a single decade the number of adult practices with unfilled GP posts doubled, against a background of steadily rising practitioner age [15]. By July 2024, 14% of GP practices were vacant, 12.8% of them chronically unfilled, while one-third of the country’s 5517 GPs were older than 65 years and the median GP age had reached 61 [16], with the mean age rising by a further 2.8 years between 2010 and 2024 [17]. Because today’s medical students constitute the future primary care workforce, their willingness to enter family medicine is decisive for closing this gap [13].

Beyond its clinical applications, telemedicine also reshapes how physicians’ work can be organised. Because a proportion of care can be delivered remotely, it enables more flexible and partly location-independent working arrangements, and physicians who practise telemedicine report better work/life balance and less emotional exhaustion than colleagues who do not [18]. This is directly relevant to the workforce problem, because work/life balance and a controllable lifestyle are among the strongest determinants of specialty choice and have historically drawn students away from primary care toward more lifestyle-oriented disciplines [19]. To the extent that telemedicine increases the flexibility and controllability of general practice, it might offset one of the field’s traditional deterrents and enhance its appeal, although whether it does so depends on how prospective entrants perceive it.

Research on medical students and telemedicine has to date concentrated on their attitudes, awareness, and often deficient knowledge; students generally hold favourable views, but their knowledge is limited, which reviews attribute to the scarcity of telemedicine teaching in undergraduate education [20,21]. Studies grounded in technology acceptance theory further indicate that the perceived usefulness of telemedicine, rather than mere familiarity, most strongly drives students’ intention to use it [22]. This research, however, has examined telemedicine competence and general attitudes, not whether students connect telemedicine to the attractiveness of a career in a particular specialty. To our knowledge, no previous study has specifically examined the relationship between telemedicine and the perceived attractiveness of a family medicine career among medical students, and evidence from Central and Eastern Europe is especially scarce. This cross-sectional questionnaire study therefore aimed to characterise Hungarian fifth-year medical students’ views of telemedicine along four dimensions: (i) their awareness and self-perceived knowledge; (ii) the perceived usefulness of specific telemedicine modalities in general practice; (iii) their perceptions of its impact on the efficiency and quality of general practice care; and (iv) whether they consider telemedicine, and the flexible or remote working arrangements it enables, to make a family medicine career more attractive, together with its perceived advantages and disadvantages. We emphasise at the outset that the outcome examined here is the perceived attractiveness of the career, not actual specialty choice.

## 2. Materials and Methods

### 2.1. Study Design and Participants

This was a cross-sectional, self-administered questionnaire study among fifth-year medical students at the University of Debrecen, Hungary. Data were collected in early 2026 (between 27 January and 13 February 2026) using a printed, paper-based questionnaire (Supplementary Materials) that participants completed on site; the responses were subsequently entered into an electronic form (Google Forms; Google LLC, Mountain View, CA, USA) for digitisation and analysis. Participation was voluntary and anonymous, and no personally identifiable information was recorded. Eligible participants were the fifth-year students of the Hungarian-language medical programme. Of the 154 students in this cohort, 140 completed the questionnaire, giving a response rate of 90.9%.

### 2.2. Questionnaire

Data were collected with a structured, self-developed questionnaire in Hungarian; the instrument was not formally validated or piloted, and its full English translation is provided as Supplementary Material. It comprised five thematic blocks. The first captured sociodemographic characteristics (sex, age, type of settlement of residence, and county) and the respondent’s interest in a family medicine career on a four-point scale. The second assessed telemedicine awareness (whether the respondent had heard of telemedicine; yes/no) and self-perceived knowledge of its forms on a five-point scale (from “not at all informed” to “very well informed”). The third block asked respondents to rate the perceived usefulness of seven telemedicine modalities in general practice, each on a five-point scale (1 = not useful at all; 5 = very useful): video (online) consultation, telephone consultation, e-mail-based physician/patient communication, use of remote diagnostic devices (e.g., analysis of home blood pressure-monitoring data), mobile-application-based health monitoring, communication via chat applications (e.g., Facebook Messenger, WhatsApp), and SMS-based communication. These modalities were chosen to represent the recognised categories of telemedicine used for clinical interaction at a distance: real-time consultation (video, telephone), asynchronous clinical communication (e-mail, chat applications, SMS), and remote data capture or monitoring (remote diagnostic devices, mobile-application monitoring) [2]; the text-based channels were framed explicitly as physician/patient clinical communication rather than general-purpose social messaging. The fourth block addressed perceptions of telemedicine’s impact on general practice: whether wider adoption would make GP work more attractive (yes/no/do not know); the perceived attractiveness of home-office/remote GP work (five-point scale, including a “do not know” option); the preferred working arrangement (in-office only, home-office only, or hybrid); and the perceived effects on the efficiency of GP work and on the quality of patient care (five-point scales). The fifth block asked respondents to select the most important perceived advantages and the greatest perceived disadvantages of telemedicine (multiple-response items) and included one optional free-text field.

Missing data were limited to a single item, mobile-application-based monitoring, which two respondents left blank; no other item had missing values. For these two participants the composite usefulness score (defined below) was computed from the remaining six items, so all 140 respondents were retained in every analysis.

### 2.3. Statistical Analysis

All analyses were performed in IBM SPSS Statistics (version 27; IBM Corp., Armonk, NY, USA). Categorical variables were summarised as frequencies and percentages. Ordinal and Likert-type variables (self-perceived knowledge, perceived usefulness of each modality, the attractiveness of home-office work, interest in a family medicine career, and the perceived effects on efficiency and quality) were treated as ordinal and summarised as medians with interquartile ranges (IQR) and full frequency distributions. The normality of age was assessed with the Shapiro–Wilk test and, being non-normal, it is reported as the median (IQR) and range. Given the ordinal nature of the attitudinal items, non-parametric methods were used throughout.

A composite perceived usefulness score was computed as the mean of the seven modality items, and its internal consistency was evaluated with Cronbach’s alpha. Because these items are not necessarily unidimensional, their structure was examined by principal component analysis (PCA), and two conceptually based subscales were defined a priori (a synchronous/clinical data subscale comprising video, telephone, remote diagnostic devices, and mobile-application monitoring; and an informal-text subscale comprising e-mail, chat applications, and SMS), each with its own Cronbach’s alpha. Bivariate associations were examined with the Pearson chi-square test (or Fisher’s exact test when any expected cell count was below five, with sparse categories collapsed a priori) for categorical variables; the Mann–Whitney U test for an ordinal outcome between two groups and the Kruskal–Wallis test across more than two groups; and Spearman’s rank correlation coefficient (rho, reported with a 95% confidence interval) between two ordinal variables. Effect sizes were reported with every test: the phi coefficient or Cramer’s V for chi-square tests, odds ratios (ORs) with 95% confidence intervals (CIs) for 2 × 2 tables, the correlation-type effect size r = Z/√N (Rosenthal’s *r*, where *N* is the number of observations in the comparison) for Mann–Whitney tests, epsilon-squared for Kruskal–Wallis tests, and rho for correlations.

To identify independent correlates of the primary outcome, the perception that wider adoption of telemedicine would make general practice work more attractive (dichotomised as “yes” versus “no/do not know”), a binary logistic regression model was fitted. Sex was coded as male versus female (reference) and age as a continuous variable in years; interest in a family medicine career (1–4), self-perceived knowledge (1–5), and the composite usefulness score (1–5) were entered as continuous, per-point terms, so their adjusted ORs express the change in odds per one-point increase. With 45 positive outcomes and five predictors the model had nine events per variable, marginally below the conventional threshold of ten; the model was therefore kept parsimonious, no stepwise selection was applied, and its estimates are interpreted cautiously. Model fit was assessed with the likelihood-ratio test, the Nagelkerke R2, and the Hosmer–Lemeshow test.

As this study was largely exploratory, a small set of primary comparisons was specified a priori: (i) the association between interest in a family medicine career and the perception that telemedicine would make GP work more attractive; (ii) the difference in the composite usefulness score by sex; (iii) the difference in the perceived attractiveness of home-office work by interest; and (iv) the correlation between self-perceived knowledge and the composite usefulness score. The family-wise error rate across these four comparisons was controlled with the Holm–Bonferroni method; all other analyses were treated as exploratory and reported with unadjusted *p*-values. Pre-planned sensitivity analyses refitted the primary model with interest dichotomised, re-analysed the outcome excluding “do not know” responses (yes versus no), and replaced the composite score with the two subscale scores. All tests were two-sided, statistical significance was set at *p* < 0.05, and exact *p*-values with 95% CIs are reported.

## 3. Results

### 3.1. Sample Characteristics

A total of 140 fifth-year medical students completed the questionnaire (response rate 90.9%). The sample was approximately balanced by sex (73 women, 52.1%; 67 men, 47.9%). Age was non-normally distributed (Shapiro–Wilk W = 0.729, *p* < 0.001) and is summarised as the median: 24 years (IQR 23–25; range 22–37). Most respondents lived in urban settlements, most commonly a town of county rank (76, 54.3%) or a town (35, 25.0%), and the majority resided in the eastern Hungarian counties of Hajdu–Bihar (69, 49.3%) and Szabolcs–Szatmar–Bereg (30, 21.4%). Interest in a family medicine career was almost evenly divided: 66 students (47.1%) were “rather” or “very” interested and 74 (52.9%) were “rather not” or “not at all” interested (median 2, IQR 2–3). Notably, the interested group was dominated by “rather interested” respondents (60 of 66), with only 6 students “very interested”. Detailed sample characteristics are given in Table 1.

### 3.2. Awareness of Telemedicine and Perceived Usefulness of Its Modalities

Awareness of telemedicine was almost universal: 138 respondents (98.6%) had previously heard of the concept and 2 (1.4%) had not. Self-perceived knowledge was modest (median 3, IQR 2–3); the full distribution was 1 respondent (0.7%) “not at all informed”, 44 (31.4%) knowing “little”, 80 (57.1%) “averagely informed”, 13 (9.3%) “rather” well informed, and 2 (1.4%) “very” well informed. The perceived usefulness of the seven modalities in general practice varied markedly (Table 2). It was highest for remote diagnostic devices (mean 4.11 +/− 0.97; median 4) and mobile-application-based monitoring (4.01 +/− 0.99), followed by telephone (3.94 +/− 1.05) and video (3.81 +/− 0.99) consultations; e-mail was intermediate (3.36 +/− 1.24), while chat applications (2.88 +/− 1.17) and, in particular, SMS (2.49 +/− 1.23) were rated least useful. Synchronous and clinical data-oriented modalities were thus consistently rated more useful than informal, asynchronous text channels.

A composite usefulness score (mean of the seven items) was 3.51 +/− 0.72 (median 3.57, IQR 3.14–4.00), with acceptable internal consistency (Cronbach’s alpha = 0.78). Principal component analysis supported this summary while confirming the structure noted above: the first component (eigenvalue 3.07, 43.6% of variance) loaded positively on all seven items, indicating a dominant general dimension, and the second component (eigenvalue 1.29, 18.3%) contrasted the informal-text channels (SMS, chat, e-mail) with the clinical data and synchronous modalities. The two a priori subscales showed acceptable internal consistency (synchronous/clinical data subscale, alpha = 0.72, mean 3.97 +/− 0.73; informal-text subscale, alpha = 0.74, mean 2.91 +/− 0.98) and were moderately correlated (r = 0.47).

### 3.3. Perceived Impact of Telemedicine on General Practice Work

When asked whether wider adoption of telemedicine would make general practice work more attractive to them, 45 students (32.1%) answered affirmatively, 60 (42.9%) said it would not, and 35 (25.0%) were undecided. Attitudes toward remote working were more favourable: 79 respondents (56.4%) considered home-office/remote general practice work “rather” or “very” attractive, 50 (35.7%) found it unattractive, and 11 (7.9%) were undecided (median 4, IQR 2–4). Preferences regarding the mode of work were strongly concentrated: 116 students (82.9%) preferred a hybrid arrangement, 20 (14.3%) favoured exclusively in-office care, and only 4 (2.9%) exclusively home-office work. Perceptions of the effect on efficiency were predominantly positive, with 106 students (75.7%) expecting telemedicine to increase or somewhat increase efficiency, 17 (12.1%) anticipating no change, and 17 (12.1%) expecting a decrease (median 4, IQR 4–4). Views on the effect on care quality were more divided: 82 respondents (58.6%) expected an improvement, 28 (20.0%) no change, and a substantial minority of 30 (21.4%) expected quality to worsen (median 4, IQR 3–4). Overall, the students viewed telemedicine favourably in terms of flexibility and efficiency and clearly preferred hybrid working, yet only about one-third regarded it as a factor that would make the GP career itself more attractive.

### 3.4. Perceived Advantages and Disadvantages of Telemedicine

Respondents could select multiple perceived advantages and disadvantages (Table 3). The most frequently endorsed advantages were time efficiency (118, 84.3%) and faster consultation (103, 73.6%), followed by reduced risk of infection (86, 61.4%), easier monitoring of chronic patients (77, 55.0%), and cost-effectiveness (76, 54.3%). Digital documentation (68, 48.6%), easier follow-up (64, 45.7%), and increased accessibility (59, 42.1%) were chosen less often, and environmental sustainability was the least frequent (38, 27.1%). Among the disadvantages, concerns centred on the clinical limitations of remote care: that telemedicine is not applicable in every situation (102, 72.9%) and offers only limited scope for physical examination (101, 72.1%) were the most common, followed by communication difficulties (73, 52.1%), technical failures (72, 51.4%), and general technological limitations (66, 47.1%). Reduced patient trust (54, 38.6%) and, notably, data security (39, 27.9%) were the least frequently selected concerns.

### 3.5. Factors Associated with Perceiving Telemedicine as Increasing the Attractiveness of GP Work

In the pre-specified, Holm–Bonferroni-corrected primary analyses, students interested in a family medicine career were more likely than those not interested to perceive telemedicine as making general practice work more attractive (29/66, 43.9% vs. 16/74, 21.6%; chi-square = 7.97, df = 1, *p* = 0.005, Holm-adjusted *p* = 0.014; Cramer’s V = 0.24; OR = 2.84, 95% CI 1.36–5.93). Interested students also rated home-office work as more attractive (median 4 vs. 2; Mann–Whitney test on the 129 respondents who gave a rating, i.e., excluding 11 “do not know” responses; Z = 3.11, *p* = 0.002, Holm-adjusted *p* = 0.007; r = 0.27). By contrast, the composite usefulness score did not differ by sex (Z = 1.09, *p* = 0.275, r = 0.09), and self-perceived knowledge showed no evidence of an association with the composite usefulness score (Spearman’s rho = 0.024, 95% CI −0.14 to 0.19, *p* = 0.775). Exploratory, unadjusted comparisons revealed no further significant associations (all *p* > 0.05).

In the binary logistic regression model (n = 140; 45 events; Table 4), interest in a family medicine career (adjusted OR = 2.59 per one-point increase, 95% CI 1.47–4.57, *p* = 0.001) and the composite usefulness score (adjusted OR = 2.11 per point, 95% CI 1.17–3.82, *p* = 0.013) were the only independent predictors; self-perceived knowledge showed a non-significant trend (OR = 1.70, 95% CI 0.94–3.07, *p* = 0.077), and sex and age were not associated. The model was significant (likelihood-ratio chi-square(5) = 23.70, *p* < 0.001), explained a moderate proportion of variance (Nagelkerke R2 = 0.22), and showed adequate calibration (Hosmer–Lemeshow *p* = 0.11).

### 3.6. Sensitivity Analyses

When interest was entered as a dichotomous term (interested versus not interested), its adjusted OR was 3.81 (95% CI 1.69–8.58, *p* = 0.001), directly comparable with, and consistent in direction and magnitude with, the bivariate OR of 2.84. Excluding the 35 “do not know” respondents (yes versus no; n = 105), interest remained a strong independent correlate (adjusted OR 3.03 per point, 95% CI 1.61–5.68, *p* = 0.001), whereas the association with the composite usefulness score attenuated to non-significance (OR 1.74, 95% CI 0.95–3.19, *p* = 0.071). When the composite was replaced by the two subscales, interest again dominated (OR 2.60, *p* = 0.001), while neither the synchronous/clinical data subscale (OR 1.62, 95% CI 0.85–3.06, *p* = 0.14) nor the informal-text subscale (OR 1.34, 95% CI 0.87–2.06, *p* = 0.19) was independently significant, suggesting that the composite’s association reflects overall perceived usefulness rather than either dimension alone. Across all sensitivity analyses, interest in a family medicine career remained the most robust correlate of the outcome.

## 4. Discussion

This cross-sectional survey of fifth-year Hungarian medical students yielded four principal findings. First, although awareness of telemedicine was near-universal, self-perceived knowledge was modest and showed no evidence of an association with how useful students judged telemedicine to be. Second, perceived usefulness was clearly structured: synchronous and clinical data modalities were rated useful, whereas informal asynchronous text channels were not. Third, students strongly preferred hybrid working and generally expected telemedicine to improve the efficiency of general practice work, though views on care quality were more divided. Fourth, only about one-third thought telemedicine would make a GP career more attractive, a view driven by pre-existing career interest and by perceived usefulness rather than by sex, age, or self-rated knowledge.

The coexistence of near-universal awareness with limited self-perceived knowledge echoes the international literature, in which reviews report predominantly favourable student attitudes alongside limited knowledge [20]; our data reproduce this pattern, with awareness of 98.6% but a median self-rated knowledge of only 3 of 5, a gap that reviews attribute to insufficient telemedicine teaching in the curriculum [20,21]. Crucially, self-rated knowledge did not predict perceived usefulness, whereas perceived usefulness itself was the principal attitudinal correlate of the career attractiveness outcome. This ordering matches technology acceptance research in medical students, in which performance expectancy, essentially perceived usefulness, is the strongest determinant of the intention to use telemedicine [22], and studies applying the Unified Theory of Acceptance and Use of Technology (UTAUT) that identify performance expectancy, trust, and self-efficacy, rather than familiarity, as the active ingredients of adoption [23]. It is consistent, too, with the finding that Hungarian primary care physicians are more receptive to communication-supporting than to clinical work-supporting tools [9], mirroring our students’ ranking of the modalities, and with evidence that the main barriers to adoption are organisational and attitudinal rather than knowledge-based [24]. Together these findings argue for embedding telemedicine competencies in undergraduate training [21], so that favourable attitudes are anchored in demonstrable skills.

The structured pattern of perceived usefulness is clinically coherent and links our descriptive findings to the wider evidence base. Modalities that transmit objective clinical data or preserve real-time interaction were rated most useful, whereas asynchronous text channels were viewed with reservation, an ordering that matches the students’ most frequently cited disadvantages (that telemedicine is not applicable in every situation and offers only limited scope for physical examination) and the evidence that roughly one-fifth of physical examination components do not translate to remote assessment [25]. The relative preference for video and telephone over text accords with comparative studies in which video consultations are at least equivalent to telephone [26,27], although both convey less information than face-to-face encounters [28]. Reassuringly for the efficiency perceptions we observed, teleconsultations achieve outcomes broadly comparable to in-person care for many primary care presentations [29], and differences between telehealth and in-person care in utilisation and outcomes are generally small and of limited clinical significance [30]. That data security ranked lowest among perceived disadvantages is notable, given the genuine privacy risks of transmitting health data over open networks [31], and may reflect either trust in institutional safeguards or limited awareness of information governance risks.

Against a well-documented primary care workforce crisis [12,13,15,16,17], the question of whether telemedicine can make general practice more attractive is of direct policy relevance. Family medicine has long been perceived as a discipline of comparatively low interest and prestige [32], and this perceived prestige is itself linked to career interest [33]; conversely, a pleasant working environment is among the strongest positive predictors of choosing general practice [34]. Work/life balance and a controllable lifestyle likewise shape specialty choice and have historically favoured other disciplines [19], whereas physicians who practise telemedicine report better work/life balance and less emotional exhaustion [18], and GPs value telemedicine more highly than colleagues in other specialties [35]. These observations make telemedicine a plausible lever for enhancing the appeal of general practice. Our data temper this expectation: only one-third of students regarded telemedicine as increasing the attractiveness of general practice, and this view was concentrated among those already interested in the field. In the regression, interest was by far the strongest correlate; with interest on its original scale the adjusted odds rose about 2.6-fold per point, and when interest was dichotomised the adjusted odds ratio (3.81) closely matched the unadjusted bivariate estimate (2.84). Because, to our knowledge, no prior study has measured this specific outcome, a direct quantitative comparison is not yet possible and our figures may serve as an initial benchmark. Above all, the outcome was perceived attractiveness, not realised specialty choice, so these associations describe attitudes that may or may not translate into workforce entry; telemedicine may therefore reinforce an existing inclination toward family medicine rather than independently recruit the undecided, a reading reinforced by our sensitivity analyses, in which the association with perceived usefulness weakened when the undecided were excluded while the association with career interest remained robust.

At the same time, the near-unanimous preference for hybrid working and the positive view of telemedicine’s effect on efficiency indicate that digitally enabled flexibility appeals in principle to the coming generation of physicians, mirroring the marked rise in Hungarian population-level telemedicine adoption between 2021 and 2024 [10]. Realising this potential in general practice will require implementation that protects the aspects of clinical work students value, particularly care quality, about which one-fifth expressed concern, and a stable regulatory and reimbursement framework of the kind established during the pandemic [5]. It will also require attention to patient-facing barriers: limited eHealth literacy and the digital divide risk widening inequities, since in Hungary those expected to benefit most from digital health tend to use it least [36], and equitable telehealth for older and vulnerable groups depends on digital literacy and connectivity [37]. Ultimately, patient acceptance itself hinges on the purpose of the consultation, its convenience, and continuity of care [38].

This study has several limitations. It was conducted at a single institution; although the response rate was high (90.9%), which limits the scope for non-response bias, participation was voluntary and the findings may not generalise to other cohorts or settings. The questionnaire was self-developed and neither formally validated nor piloted, and knowledge was self-reported rather than objectively assessed. Most importantly, we assessed the perceived attractiveness of a family medicine career, not actual specialty choice; perceptions and stated intentions are imperfect proxies for eventual career decisions, and only longitudinal designs that link attitudes to realised choices can establish whether telemedicine influences workforce entry. The interested group consisted mostly of “rather interested” students, so the career interest finding should be read as a modest inclination rather than strong commitment. The cross-sectional design precludes causal inference, and the primary outcome was a single item with a “do not know” option that we grouped with negative responses; the sensitivity analyses support the robustness of the career interest finding to this choice, but the undecided may differ systematically from those who declined. Social-desirability and non-response biases cannot be excluded, and, with nine events per variable, the regression was necessarily parsimonious. Finally, analyses beyond the pre-specified comparisons were exploratory and should be regarded as hypothesis-generating.

## 5. Conclusions

Among fifth-year Hungarian medical students, telemedicine was familiar and broadly regarded as useful, especially in its synchronous and clinical data forms, and hybrid working was strongly preferred. Yet only a minority regarded telemedicine as a factor that would make a general practice career more attractive, and this perception was tied to pre-existing career interest and perceived usefulness rather than to demographic characteristics or self-rated knowledge. Telemedicine should therefore be seen as a complement to, rather than a substitute for, broader measures to make family medicine attractive, and its promise for the primary care workforce will depend on integrating telemedicine competencies into the curriculum and on implementation that safeguards care quality. Framed within the broader agenda of primary care informatics and digital health, in which adoption is driven by digital literacy, motivated staff, and adequate resourcing [39], these findings bear directly on training, education, and workforce development in digital health. Because this study measured perceived attractiveness rather than realised career choice, multi-centre and longitudinal studies are warranted to confirm these findings and to track how attitudes translate into workforce entry as telemedicine becomes embedded in routine practice.

## Figures and Tables

**Table 1:** Characteristics of the study sample (N = 140).

Characteristic	Category	n (%)
Sex	Female	73 (52.1)
	Male	67 (47.9)
Age, years	Median (IQR); range	24 (23–25); 22–37
Settlement of residence	Town of county rank	76 (54.3)
	Town	35 (25.0)
	Village	19 (13.6)
	Large village	5 (3.6)
	Capital (Budapest)	5 (3.6)
County	Hajdu-Bihar	69 (49.3)
	Szabolcs–Szatmar–Bereg	30 (21.4)
	Borsod–Abauj–Zemplen	14 (10.0)
	Heves	10 (7.1)
	Budapest	5 (3.6)
	Pest	5 (3.6)
	Jasz–Nagykun–Szolnok	3 (2.1)
	Bekes	3 (2.1)
	Nograd	1 (0.7)
Interest in a family medicine career	Very interested	6 (4.3)
	Rather interested	60 (42.9)
	Rather not interested	52 (37.1)
	Not at all interested	22 (15.7)

**Table 2:** Perceived usefulness of telemedicine modalities in general practice (N = 140), rated from 1 (not useful at all) to 5 (very useful).

Modality	Median (IQR)	Mean +/− SD
Remote diagnostic devices (e.g., home BP-monitoring data)	4 (4–5)	4.11 +/− 0.97
Mobile-application-based health monitoring a	4 (3–5)	4.01 +/− 0.99
Telephone consultation	4 (3–5)	3.94 +/− 1.05
Video (online) consultation	4 (3–5)	3.81 +/− 0.99
E-mail-based communication	3 (2–4)	3.36 +/− 1.24
Communication via chat applications	3 (2–4)	2.88 +/− 1.17
SMS-based communication	2 (1–3)	2.49 +/− 1.23
Composite usefulness score b	3.57 (3.14–4.00)	3.51 +/− 0.72

**Table 3:** Perceived advantages and disadvantages of telemedicine (N = 140; multiple responses permitted).

Perceived Advantage	n (%)	Perceived Disadvantage	n (%)
Time efficiency	118 (84.3)	Not applicable in every situation	102 (72.9)
Faster consultation	103 (73.6)	Limited examination possibilities	101 (72.1)
Reduced risk of infection	86 (61.4)	Communication difficulties	73 (52.1)
Easier monitoring of chronic patients	77 (55.0)	Technical failures	72 (51.4)
Cost-effectiveness (travel, time, infrastructure)	76 (54.3)	Technological limitations	66 (47.1)
Digital documentation	68 (48.6)	Reduced patient trust	54 (38.6)
Easier follow-up	64 (45.7)	Data security	39 (27.9)
Increased accessibility	59 (42.1)	(not applicable)	-
Environmental sustainability	38 (27.1)	(not applicable)	-

**Table 4:** Binary logistic regression predicting the perception that telemedicine would make general practice work more attractive (yes vs. no/do not know); N = 140. Ordinal predictors were entered as continuous per-point terms.

Predictor	Coding	B (SE)	*p*	OR (95% CI)
Interest in family medicine career	per 1 point (1–4)	0.953 (0.289)	0.001	2.59 (1.47–4.57)
Telemedicine usefulness (composite)	per 1 point (1–5)	0.748 (0.302)	0.013	2.11 (1.17–3.82)
Self-perceived knowledge	per 1 point (1–5)	0.532 (0.301)	0.077	1.70 (0.94–3.07)
Sex	male vs. female	0.577 (0.410)	0.160	1.78 (0.80–3.98)
Age	per year	0.127 (0.108)	0.242	1.14 (0.92–1.40)

Model: likelihood-ratio chi-square(5) = 23.70, *p* < 0.001; Nagelkerke R2 = 0.22; Hosmer–Lemeshow *p* = 0.11; events per variable = 9. OR, odds ratio; CI, confidence interval.

## Data Availability

The data presented in this study are not publicly available. The informed consent obtained from participants covered the use of their anonymised data by the research team for its own research and publications only and did not include provisions for transferring the dataset to third parties or making it publicly available, even in anonymised form. In addition, given the small, single-institution sample, the combination of demographic variables could entail a risk of re-identification. In line with this study’s ethical approval and with GDPR requirements, the data may be made available by the corresponding author on reasonable request, subject to approval by the Regional and Institutional Research Ethics Committee of the University of Debrecen and to a data-sharing agreement.

## Supplementary Materials

The following supporting information can be downloaded at https://www.mdpi.com/article/10.3390/healthcare14193256/s1, Questionnaire S1: English translation of the self-developed questionnaire on fifth-year medical students’ attitudes toward telemedicin.

## Abbreviations

The following abbreviations are used in this manuscript:

CI	Confidence interval
GP	General practitioner
IQR	Interquartile range
OR	Odds ratio
PCA	Principal component analysis
UTAUT	Unified Theory of Acceptance and Use of Technology
WHO	World Health Organization
