# Supplementary material for: Telemedicine and the Attractiveness of a Family Medicine Career Among Hungarian Medical Students: Implications for the Digital Health Primary Care Workforce
Source: Healthcare (Basel). 2026 Oct 1;14(19):3256. doi: 10.3390/healthcare14193256 (PMC13664772; doi:10.3390/healthcare14193256)
Supplement: Supplementary file 1 [file healthcare-14-03256-s001.zip › healthcare-4594677-supplementary.pdf]

Supplementary Material. Study questionnaire (English translation)

Original instrument in Hungarian; translated for publication. Response options are listed; free-text and "other" fields are indicated.

Section A. Demographic questions

Sex:

- Male
- Female
- Other

Age: ____

Place of residence:

- Capital city
- Town of county rank
- Town
- Large village
- Village

County of residence: ____

Are you interested in a career as a general practitioner (family physician)?

- Very interested
- Rather interested
- Rather not interested
- Not at all interested

Section B. Questionnaire on telemedicine

1. Have you heard of the concept of telemedicine?

- Yes
- No

2. If yes, how well informed do you feel about the various forms of telemedicine?

- Not at all
- I know little about it
- I am averagely informed
- I am rather well informed

- I am very well informed

3. Please rate the following forms of telemedicine according to how useful you consider them in general-practice work (1 = not useful at all, 5 = very useful):

**Form**	**1**	**2**	**3**	**4**	**5**
Online consultation (video call)					
Telephone consultation					
E-mail-based physician-patient communication					
Use of remote diagnostic devices (e.g., analysis of home blood-pressure monitor data)					
Mobile-application-based health monitoring					
Communication via chat applications (e.g., Facebook Messenger, WhatsApp)					
SMS-based physician-patient communication					

Section C. Telemedicine and the general-practice career

Would wider use of telemedicine make general-practice work more attractive to you?

- Yes
- No
- I do not know

Do you find general-practice work performed as home office / remote work attractive?

- I would find it very attractive
- I would find it rather attractive
- I do not know
- I would find it rather unattractive
- I would find it not attractive at all

Which mode of work would be most attractive to you as a general practitioner?

- In-office patient care only
- Home-office work only
- Hybrid (combination of in-person and home office)
- Other: ____

In your opinion, how does the use of telemedicine affect the efficiency of general-practice work?

- Decreases it
- Rather decreases it
- Neutral
- Rather increases it
- Increases it

In your opinion, how does telemedicine affect the quality of patient care?

- Worsens it
- Rather worsens it
- Neutral
- Rather improves it
- Improves it

In your opinion, what are the most important advantages of telemedicine? (Multiple answers possible)

- Time efficiency
- Increased accessibility
- Faster consultation
- Easier follow-up of chronic patients
- Reduced risk of infection
- Cost-effectiveness (travel, time, infrastructure)
- Digital documentation
- Easier follow-up
- Environmental awareness, smaller ecological footprint
- Other: ____

In your opinion, what are the greatest disadvantages of telemedicine? (Multiple answers possible)

- Technological limitations
- Limited examination possibilities
- Communication difficulties
- Data security
- Not applicable in every situation
- Technical failures
- Reduced patient trust
- Other: ____

If you have any further thoughts, opinions, or observations about the use of telemedicine in general-practice work, please describe them here: (free text)
